# Handling linkage disequilibrium in linkage analysis using dense single-nucleotide polymorphisms

**DOI:** 10.1186/1753-6561-1-s1-s161

**Published:** 2007-12-18

**Authors:** Kelly Cho, Qiong Yang, Josée Dupuis

**Affiliations:** 1 Department of Biostatistics, Boston University School of Public Health, 715 Albany Street, Talbot E 4th Floor, Boston, Massachusetts 02118, USA

## Abstract

The presence of linkage disequilibrium violates the underlying assumption of linkage equilibrium in most traditional multipoint linkage approaches. Studies have shown that such violation leads to bias in qualitative trait linkage analysis when parental genotypes are unavailable. Appropriate handling of marker linkage disequilibrium can avoid such false positive evidence. Using the rheumatoid arthritis simulated data from Genetic Analysis Workshop 15, we examined and compared the following three approaches to handle linkage disequilibrium among dense markers in both qualitative and quantitative trait linkage analyses: a simple algorithm; SNPLINK, methods for marker selection; and MERLIN-LD, a method for modeling linkage disequilibrium by creating marker clusters. In analysis ignoring linkage disequilibrium between markers, we observed LOD score inflation only in the affected sib-pair linkage analysis without parental genotypes; no such inflation was present in the quantitative trait locus linkage analysis with severity as our phenotype with or without parental genotypes. Using methods to model or adjust for linkage disequilibrium, we found a substantial reduction of inflation of LOD score in affected sib-pair linkage analysis. Greater LOD score reduction was observed by decreasing the amount of tolerable linkage disequilibrium among markers selected or marker clusters using MERLIN-LD; the latter approach showed most reduction. SNPLINK performed better with selected markers based on the D' measure of linkage disequilibrium as opposed to the *r*^2 ^measure and outperformed the simple algorithm. Our findings reiterate the necessity of properly handling dense markers in linkage analysis, especially when parental genotypes are unavailable.

## Background

With rapid development of high-throughput genotyping technologies, more researchers have begun genome-wide searches for genes associated with complex diseases using dense single-nucleotide polymorphisms (SNPs). These dense SNPs create clusters of SNPs in linkage disequilibrium (LD) along each chromosome. The underlying assumption of linkage equilibrium (LE) between markers in many multipoint linkage methods is violated among dense SNPs where LD may exist. Such violation can lead to incorrect pedigree haplotype inference [1], especially with missing parental genotypes, resulting in bias in linkage analysis. Huang and colleagues [2] have shown that LE assumption among tightly linked markers induces false-positive evidence for linkage in qualitative trait linkage analysis with missing parental genotypes. This bias may be influenced by SNPs in LD, which can cause apparent oversharing of multipoint identity by descent (IBD). Thus, appropriate LD modeling or adjusting for markers in LD is needed to avoid potential bias in linkage in the case of missing parental genotypes. In our study, we sought to examine and compare three different approaches of handling LD in both affected sib-pair (ASP) and quantitative trait locus (QTL) linkage analysis using dense SNPs on chromosome 6 from the Genetic Analysis Workshop 15 (GAW15) simulated data without prior knowledge of the answers. The methods include a simple algorithm, SNPLINK [3], and MERLIN-LD [4].

## Methods

### Data and selection of genetic region

Rheumatoid arthritis (RA) simulated data provided by GAW15 include 1500 pedigrees (nuclear families, two parents, and two offspring) with 6000 individuals genotyped with a very dense set of 17,820 SNPs on chromosome 6. We focused our analysis on a sub-region containing 937 SNPs along chromosome 6 (170 to 185 cM) where there is no evidence for linkage. Our selection was based on the availability of dense SNPs on chromosome 6 combined with our attempt to find a null region that was farthest from the strong linkage peak at 49 cM to evaluate false-positive evidence for linkage by inflated LOD scores. Each pedigree data set was modified to create two additional data sets with one and both parental genotypes missing for the purpose of evaluating false-positive linkage with ungenotyped parents. According to the distribution of the LD measures, the following sets of LD thresholds by D' and *r*^2 ^were applied in handling LD: 0.1, 0.3, 0.5, and 0.7 for D' and 0.01, 0.1, 0.3, and 0.5 for *r*^2^. Using varying cut points served our goal to show a gradual change or trend of LOD score inflation along all ranges of LD measures. We performed linkage analysis over all 100 replicates of the simulated data at different LD thresholds and three pedigree data sets, with zero, one or two ungenotyped parents, and compared our results to the unadjusted linkage signal obtained using the complete marker data with no ungenotyped parents.

### ASP and QTL linkage analyses

The RA simulated data consist of families with one ASP. We used nonparametric linkage (NPL) analysis implemented in MERLIN [5] to evaluate linkage. NPL LOD scores were computed using the Kong and Cox linear model based on all affected pedigree members [6]. RA simulated data also provide phenotype variables including severity category; however, the data were unascertained with respect to this variable. The severity category variable contained ordinal values ranging from 1 to 5, where 5 refers to the most severe RA condition. Treating this severity category variable as a quantitative trait, we performed QTL linkage analysis using MERLIN-regress [7] and our implementation of a robust score statistic [8].

### Simple algorithm

Based on the selected sub-region, pairwise LD (D' and *r*^2^) was calculated using Haploview [9] from the control data. The simple algorithm adjusts LD by removing SNPs. Among the set of SNP pairs above a given threshold, the more informative SNP (i.e., the SNP with a higher minor allele frequency) is retained from each SNP pair, and all the other SNPs that are paired with the less informative SNP are then removed from the set. This procedure was repeated iteratively until no pairwise LD measure exceeded the threshold.

### SNPLINK

SNPLINK consists of a Perl Script that interfaces with other available linkage software such as MERLIN to undertake automated analyses and addresses the issue of LD [3]. LD is handled by keeping one SNP from each set of markers in LD. A set is defined where each consecutive marker pair in the set is found to be in LD at the specified threshold. From each set, the middle SNP is retained. A new LD-reduced set of SNPs is used in linkage analysis. The marker selection can be based on two LD measure, D' or *r*^2^.

### MERLIN LD

MERLIN has a built-in option of modeling LD (-rsq) by organizing markers into clusters using pre-specified *r*^2 ^[4]. MERLIN then uses population haplotype frequencies or available data to infer LD within each cluster. MERLIN calculates pairwise *r*^2 ^between neighboring markers and creates a cluster joining markers for which pairwise *r*^2 ^exceeds a pre-specified threshold and all intervening markers. We only evaluated linkage using *r*^2 ^because a D' option is currently unavailable.

## Results

**Figure 1:**
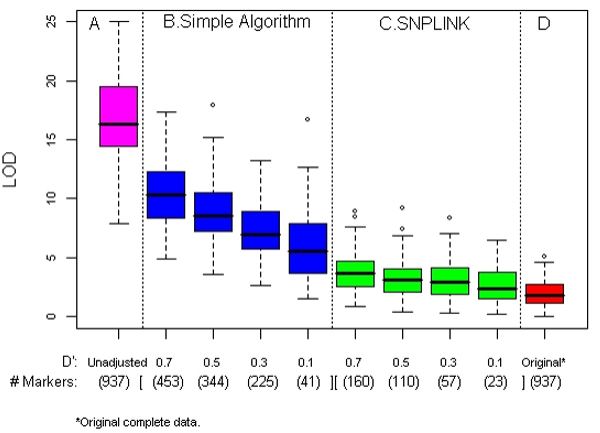
**ASP analysis: average maximum LOD score and average number of markers (D')**. Summary results from all 100 replicates with families without parental genotypes (except for the last panel, which shows the results from the complete data with full parental genotypes). Maximum LOD scores are shown in box plots with four panels: A, unadjusted, data with missing parental genotypes (pink); B, simple algorithm, with four D' thresholds (blue); C, SNPLINK, with four D' thresholds (green); and D, complete data unadjusted (red).

**Figure 2:**
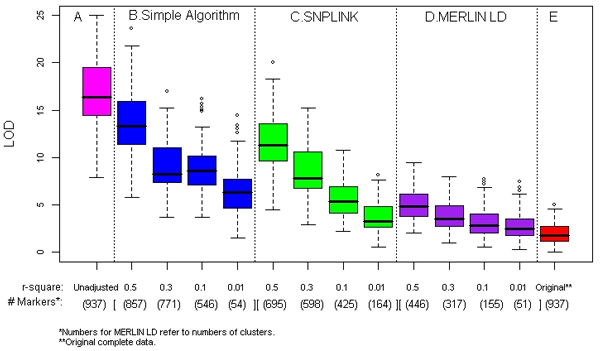
ASP analysis: average maximum LOD score and average number of markers* (*r*^2^). Summary results from all 100 replicates with families without parental genotypes (except for the last panel, which shows the results from the complete data with full parental genotypes). Maximum LOD scores are shown in box plots with five panels: A, unadjusted, data with missing parental genotypes (pink); B, simple algorithm, with four *r*^2 ^thresholds (blue); C, SNPLINK, with four *r*^2 ^thresholds (green); D, MERLIN LD, with four *r*^2 ^thresholds (purple); and E, complete data unadjusted (red).

Using the original data with complete parental genotypes, we observed no substantial linkage evidence (average maximum LOD = 2.0, SD = 1.2) over the selected sub-region in ASP linkage analysis of the RA dichotomous phenotype (last panels in Figures 1 and 2). However, ignoring LD combined with missing parental genotypes, we observed LOD score inflation shown in the panel A of Figures 1 and 2 (average maximum LOD = 17.1, SD = 3.6). Even with just one ungenotyped parent, large LOD scores were observed [average maximum of 8.5 (SD = 2.7)]. In contrast, no such inflation was observed when ignoring LD using either MERLIN-regress or the robust score statistic in QTL linkage analysis; the average maximum LOD scores for the two approaches were 0.3 and 0.2, respectively, without parental genotypes. Consequently, no inflation in LOD score was observed regardless of approaches and conditions applied in QTL analysis. Thus, we only present results from ASP linkage analysis in what follows. In general, with complete parental genotype information, all methods for handling LD with various LD thresholds yielded maximum LOD scores similar to the original linkage results (last panels in Figures 1 and 2).

As a note, in this selected sub-region, the mean, median, and mode of D' are 0.13, 0.06, and 1.0, respectively. On the other hand, most pairwise *r*^2 ^values are below 0.01 (median and interquartile range of 0.001 and 0.002, respectively). Of the 438,516 SNP pairs formed by 937 SNPs, there were 28,703 SNP pairs on average with *r*^2 ^greater than 0.01. Consequently, this resulted in most SNPs being omitted from the analysis after applying the algorithms at such threshold. In general, the number of SNPs included in each analysis decreased as the cut points decreased. In some cases, especially with the lower cut points, only 20 to 50 SNPs remained to be analyzed within our selected 15-cM region; however, this still offered a fairly dense coverage (1 to 3 SNPs per cM) and served our attempt to present a trend and magnitude of LOD score inflation due to LD among dense SNPs.

### Simple algorithm

Figures 1B and 2B show linkage analysis results using the simple algorithm at each cut point with ungenotyped parents. We observed substantial reduction of the inflated LOD score, especially with lower cut points. The average of the maximum LOD score over the 100 replicates ranges from 10.5 (SD = 2.8) at D' of 0.7 with 453 SNPs to 5.9 (SD = 2.8) at D' of 0.1 with 41 SNPs (Figure 1B). A similar pattern of reduced LOD score inflation of was observed with *r*^2 ^cut points (Figure 2B). The range of the average maximum LOD score was 6.5 (SD = 2.7), with an average 54 SNPs and 13.9 (SD = 3.4) with an average of 857 SNPs. The simple algorithm performed better with D' threshold as indicated by lower amount of LOD score inflation in this region of no linkage.

### SNPLINK

Using SNPLINK with ungenotyped parents, a similar trend of reduction of LOD score inflation was observed with lowest D' cut point showing the greatest reduction (Figure 1C). However, the average maximum LOD scores across all cut points fell in a narrow range of 2.9 (SD = 1.5) and 3.9 (SD = 1.7) for D' of 0.1 and 0.7, respectively. At the highest D', an average of 160 SNPs were evaluated. Using *r*^2 ^threshold shown by Figure 2C, such reduction of LOD score inflation with decreasing cut points remained, but with a wider range of average maximum LOD scores of 3.7 (SD = 1.6) and 11.7 (SD = 3.1). At *r*^2 ^cut point of 0.01, an average of 164 SNPs were evaluated compared to 695 SNPs at the highest cut point. SNPLINK performed better with D' threshold, as was observed for the simple algorithm.

### MERLIN LD

Figure 2D shows summary ASP linkage results using MERLIN-LD from families with no parental genotypes. We observed a tangibly reduced LOD score inflation across all cut points compared to the unadjusted (Figure 2A) and other approaches (Figure 2B and 2C). The range of average maximum LOD scores was 2.8 (SD = 1.5), with 51 clusters at *r*^2 ^= 0.01 and 5.3 (SD = 1.8) with 446 clusters at *r*^2 ^= 0.5 evaluated in linkage analysis. Although the lowest average maximum LOD score was below 3, it was still slightly higher than the value of 1.9 obtained with full parental information shown in Figure 2E.

## Conclusion

We examined and compared three approaches of handling LD using dense SNPs on chromosome 6 from the GAW15 simulated data. We performed ASP and QTL linkage analyses on a selected sub-region with low evidence of linkage (170 to 185 cM) that contains 937 SNPs. We observed inflation of LOD scores with missing parental genotypes only in ASP linkage analysis of the RA phenotype. In QTL linkage analysis, we observed no inflation in LOD scores even with missing parental genotypes using the unascertained data with respect to severity categories.

In ASP analysis across the LD thresholds (D' or *r*^2^), all three methods showed a similar pattern of less LOD score inflation as the LD cut points decreased. Using *r*^2 ^threshold, MERLIN-LD outperformed other approaches across all cut points; however, the reduction by the lowest threshold did not quite reach the LOD score with full parental genotypes available. With D' threshold, both SNPLINK and simple algorithm performed at a similar level; however, SNPLINK showed further reduction of LOD scores, especially with lower cut points. SNPLINK and the simple algorithm performed better with D' with low *r*^2^, but this result may not be generalizable to regions with high *r*^2^.

Overall, MERLIN-LD approach worked well even with the skewed *r*^2 ^distribution observed in the selected region in our sample. However, because clusters are determined based on *r*^2^, its usage is limited if D' is a more appropriate measure of LD. In addition, this approach assumes no recombination within clusters and no LD between clusters. SNPLINK appears to be flexible, with the option of using D', *r*^2^, or both combined. However, implementation takes some preparation and modification of the script is challenging. The simple algorithm is intuitive to implement but the results still contained highest inflated LOD scores at the lowest cut points compared to other approaches.

In measuring LD, *r*^2 ^provides the correlation between two SNPs and depends on the allele frequency and D' captures the ancestral history over time. Even with low *r*^2^, large D' will create bias in the probability of IBD sharing, which may lead to LOD score inflation. Therefore, applying D' threshold will remove more SNPs in handling of LD because it is generally larger than *r*^2^. In our selected region, small *r*^2 ^values reflect negligible measures of LD in practice, and thus D' measures appear to be more applicable in the analysis.

The resulting inflation with missing parental genotypes could be partially due to the decreased number of SNPs being analyzed because the information content is higher with more available parental genotypes. However, comparing the results from unadjusted data without parental data (Figs. 1A and 2A) and the original complete data (Figs. 1D and 2E) with the same number of SNPs analyzed, we see that increased information content with complete data resulted in no inflation in our study. Thus, it is more plausible to believe that the inflation is mainly due to LD in this sample rather than the decreased information content. Further, removing large number of SNPs and thus reducing the information content may affect the location accuracy and power of linkage mapping.

In conclusion, our findings reiterate the importance of properly modeling or adjusting for LD among dense markers because it gives false evidence of linkage when the parental genotypes are missing. In cases with missing parental genotypes in ASP analysis, all three approaches of adjusting for LD substantially reduced the false-positive linkage signals. The results from our study further bolster efforts to improve and develop approaches to handle linkage disequilibrium in linkage analysis using dense markers.

## Competing interests

The author(s) declare that they have no competing interests.

## References

[B1] Schaid DJ, McDonnell SK, Wang L, Cunningham JM, Thibodeau SN (2002). Caution on pedigree haplotype inference with software that assumes linkage equilibrium. Am J Hum Genet.

[B2] Huang Q, Shete A, Amos CI (2004). Ignoring linkage disequilibrium among tightly linked markers induces false-positive evidence of linkage for affected sib pair analysis. Am J Hum Genet.

[B3] Webb EL, Sellick GS, Houlston RS (2005). SNPLINK: multipoint linkage analysis of densely distributed SNP data incorporating automated linkage disequilibrium removal. Bioinformatics.

[B4] Abecasis GR, Wigginton JE (2005). Handling marker-marker linkage disequilibrium: pedigree analysis with clustered markers. Am J Hum Genet.

[B5] Abecasis GR, Cherny SS, Cookson WO, Cardon LR (2002). Merlin-rapid analysis of dense genetic maps using sparse gene flow trees. Nat Genet.

[B6] Kong A, Cox NJ (1997). Allele-sharing models: LOD scores and accurate linkage tests. Am J Hum Genet.

[B7] Sham PC, Purcell S, Cherny SS, Abecasis GR (2002). Powerful regression-based quantitative-trait linkage analysis of general pedigrees. Am J Hum Genet.

[B8] Tang H-K, Siegmund D (2001). Mapping quantitative trait loci in oligogenic models. Biostatistics.

[B9] Barrett JC, Fry B, Maller J, Daly MJ (2005). Haploview: analysis and visualization of LD and haplotype maps. Bioinformatics.

